# Concentrations of Polybrominated Diphenyl Ethers (PBDEs) in Water from Asunle Stream, Ile-Ife, Nigeria

**DOI:** 10.3390/toxics5020013

**Published:** 2017-06-16

**Authors:** Godwin O. Olutona, John A. O. Oyekunle, Aderemi O. Ogunfowokan, Olalekan S. Fatoki

**Affiliations:** 1 Department of Chemistry and Industrial Chemistry, Bowen University, Iwo 232102, Nigeria; 2 Department of Chemistry, Obafemi Awolowo University, Ile-Ife 220282, Nigeria; oyekunle@oauife.edu.ng (J.A.O.O.); remiogunfowokan@gmail.com (A.O.O.); 3 Department of Chemistry, Faculty of Applied Sciences, Cape Peninsula University of Technology, Cape Town 7530, South Africa; fatokio@cput.ac.za

**Keywords:** polybrominated diphenyl ethers, stream, concentrations, persistent organic pollutants, waste electronic and electrical equipment

## Abstract

This study assessed the concentrations of polybrominated diphenylethers (PBDEs) in stream water obtained from Asunle stream, an adjoining stream of the Obafemi Awolowo University dumpsite. Water samples were collected for a period of eight months from six different locations comprising of a spot upstream in an uphill area relative to the refuse dumpsite and five others downstream along the stream course. The sampled waters were extracted with dicholoromethane using liquid-liquid extraction method and cleanup was carried out with silica gel. The final extracts after concentration were analyzed using GC-MS/MS. The recovery experiments were adequate (105%–110%). The mean levels of Ʃ_6_PBDEs compounds analyzed ranged from 0.03 to 0.45 ng/mL. Seasonal variability of PBDEs indicated that higher levels were found during the wet season. The levels of PBDEs recorded in this work were relatively lower compared to the values reported in the literature from other developed nations.

## 1. Introduction

A critical chemical analysis of some matrices can lead to an unambiguous clarification of their pollution status. For fresh water stream systems, both the stream water and bed sediments, to varying degrees, act as important sinks of different contaminants in the freshwater system [1,2,3]. The surface water quality is strong within a given specific area and reflects the activities going on around the catchment of the surface water [4]. Small streams may carry clear water for most parts of the year whereas, during the rainy season, the water may carry a moderate amount of dirt, organic debris, and suspended materials [5]. As streams move close to inhabited or dumpsite areas, water quality can undergo various forms and levels of deterioration capable of eroding the availability of water for humans and ecosystems and even decrease species diversity within a given ecosystem [6].

Polybrominated diphenylethers are chemical compounds that have been extensively used for several decades in industries as flame retardant additives in combustible materials, such as building and textile materials, paints, mobile and computer sets, television sets, upholstery, electrical appliances, plastics and thermosetting products, interior decoration in cars, and airplanes [7,8]. The particular aim of using PBDEs in these materials is to prevent or slow down their burning process in cases of fire outbreak with the intention of protecting lives in addition to minimizing destruction and losses due to fire incidence. The bromine content of PBDEs released at the burning temperature react with the free radicals or highly oxidizing agents in the gaseous phase and consequently inhibits the ignition and burning processes. Generally, halogens are good at trapping free radicals, and thus can effectively reduce the strength of a propagating fire [9].

Polybrominated diphenylethers can be leached into the environment as a result of disposal when polymeric substances or products with sizeable quantities of PBDEs are exposed to environmental conditions that support their leaching. Many studies have shown the presence of PBDEs at significant levels in water, soil, sediment, wildlife, aquatic biota, air, dust, tissue, serum and human breast milk [7,10,11,12]. Regardless of the fire inhibiting potential of PBDEs, the impact on individual health of the chemical compounds emanating from PBDEs is a subject of immense concern in recent years. This is because these compounds and their metabolites have been discovered to be persistent, lipophilic, and noxious, and have the ability to both bioaccumulate and biomagnify, and undertake long-range atmospheric transportation [13]. Polybrominated diphenylethers can cause endocrine disruption, immunotoxicity, and developmental and reproductive dysfunctions [14,15]. The majority of these endocrine disruptors have been predicted to be in the dissolved aqueous phase because of the greater surface volume in the water body [16]. The presence of endocrine disruptors, such as PBDEs, has a great consequence on the aquatic biota. Humans are exposed to these brominated flame retardants (BFRs) released from many sources on a daily basis, principally from treated consumer products [7,9] and the contamination of human consumables by BFRs from various sources.

Investigation of PBDEs in environmental matrices such as sediment, air, and several aquatic organisms with significant concentrations and their bioaccumulation has been widely reported in the literature; for instance, in sediment [12,17,18,19], molluscs [18,20], fish [21], and water [3,20]. The hydrophobic properties of PBDEs as well as the lack of standardized methods of assessment have initially made results of their assessment in water bodies scarce in the literature, unlike in the atmospheric samples in which their seasonal variability assessments have been widely reported and documented in literature [22,23]. However, the rising apprehension about the occurrence of PBDEs in all aspects of the ecosystem has boosted the number of publications on this subject [24]. 

Taking into account their physicochemical and toxicological characteristics as enumerated in numerous studies, PBDEs are characterized as endocrine disrupting compounds capable of affecting the thyroid hormone, for example [25,26]. Endocrine disruptors are exogenous substances that interfere with the synthesis, secretion, transport, binding, action, or elimination of natural hormones that are responsible for development, behavior, fertility, and the maintenance of homeostasis in the body [27]. Polybrominated diphenylethers have been found to cause malfunctioning of intracellular second messengers. For instance, Ca^2+^ homeostasis in neuronal cells, protein kinase C, and inositol phosphate, which plays an important function in normal cellular physiology and neuronal growth [28]; and are capable of releasing arachidonic acid in cerebella granule cells through the activation of the phosphate A2 pathway, which is linked with learning and memory processes [29]. In addition to the thyroid hormone disruption caused by PBDEs, they are also suspected to be carcinogenic [30]. These and other effects of PBDEs resulted in the incorporation of PBDEs in the global treaty and concerns for water quality [24].

Generally, in Nigeria there is a dearth of scientific reports on the occurrence and concentrations of PBDEs in various environmental matrices. With the aim of protecting our water bodies, data on the distribution, fate, and current state of PBDEs in different environmental matrices are necessary to adequately assess action toward their control, monitoring, and/or elimination before it becomes a threat to both human and aquatic life. The Asunle Stream has been at the receiving end of potential pollutants, such as potentially toxic metals and various trace organic pollutants which are caused by leaching from the dumpsite. Agrochemicals used by farmers who engaged in farming activities along the stream also contribute to the source of this pollution. 

This study was aimed at assessing the concentrations of some PBDE compounds in the water of Asunle stream, an adjoining stream of the Obafemi Awolowo University, Ile-Ife dumpsite with the goal of evaluating the extent of contamination of the aquatic ecosystem with BFRs.

## 2. Materials and Methods

### 2.1. Study Area

**Figure 1:**
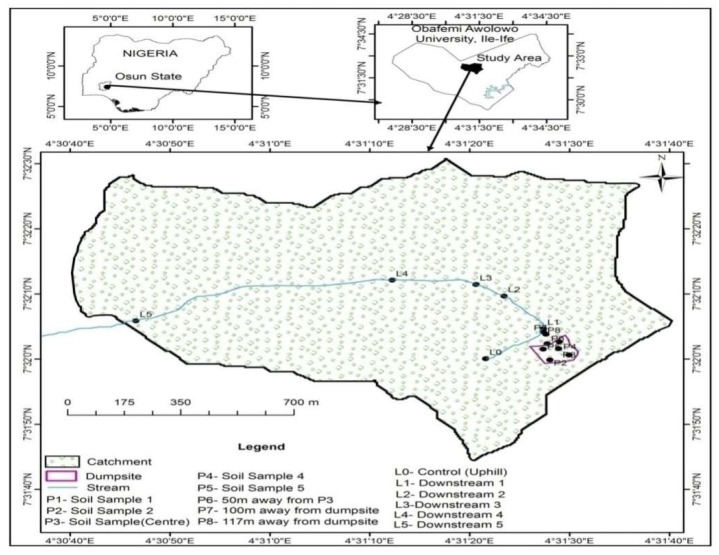
A study map of the Obafemi Awolowo University dumpsite, Ile-Ife, showing the sampling locations.

Asunle stream is a recurrent stream that has its source situated about 0.25 km uphill from the refuse dumpsite of the University (Figure 1). The stream runs a distance of more than ten kilometers, and transverses three villages [31]. Rigorous farming activities take place along the stream course which involve the planting of both cash and food crops, which serve as a source of revenue to the inhabitants of the villages within the catchment of the stream. The rural dwellers who settled close to the course of the stream use the water for household purposes, while farmers within the vicinity of the stream use the stream water for wetting their vegetable nursery beds, palm oil processing, and for dilution and spraying of agrochemicals on the cash crops planted around the area. The full description of the study area has been given by Olutona et al. (2016) [12].

### 2.2. Reagents and Chemicals

Dichloromethane (DCM), n-hexane, and isooctane used in this study were obtained from Merck, while sodium sulphate (anhydrous), sodium hydroxide, sulphuric acid, and silica gel (60–200 mm) were obtained from Sigma Aldrich, South Africa, respectively. Pentachloronitrobenzene and 50 μg/mL individual PBDE standards were purchased from Cambridge Isotope Laboratories, USA through Industrial Analytical Pty, South Africa. Helium (99.99%) and argon were obtained from Afrox Pty., South Africa.

### 2.3. Sample Collection, Preparation, and Instrumental Analysis

Sampling was conducted in the period from 2012–2013 and included two consecutive seasons—dry (November, January, and February) and wet (May–August). The monitoring program was performed on a monthly basis and samples of water were obtained from six different spots: Location 0 (control), situated upstream in an uphill area relative to the refuse dumpsite, is the source of the stream; Site 1 is near the dumpsite, about 150 m away from the dumpsite; Sites 2 to 5 are downstream locations that traverse through the agricultural farmlands and villages. Water samples could not be collected in December because the stream dried up during this period. Two and half (2.5) liter amber colored bottles were used to collect the water samples and were kept at 4 °C prior to analysis. Water samples were acidified immediately after collection in order to inhibit microbial activities and prevent the alteration of the trace organics.

One thousand mL of water was transferred into a thoroughly cleaned 2 L separatory funnel. Thirty (30) mL dichloromethane (DCM) was added to the water and was shaken vigorously for about 15 min with occasional venting over the course of each extraction. The liquid mixture was allowed to settle for about fifteen minutes for phase separation. The trace organic portion was decanted and allowed to pass through Na_2_SO_4_ in order to trap traces of water molecules in the sample and was collected in a round bottomed flask. Two other batches of extraction were repeated with 30 mL of DCM on each batch. Ten mL of DCM was used twice to rinse the separatory funnel after elimination of the aqueous layer. The collected extract was then concentrated to about a 1 mL volume.

Extracts obtained from the separatory funnel were cleaned on a column containing different types of silica. Packing of the column was carried out using multilayer silica gel chromatography. The tip of the column was first clogged with cotton wool. Silica gel was initially activated after heating to 150–160 °C. Sequential addition of 0.1 g activated silica, 0.2 g basic silica, 0.4 g acidic silica, 0.1 g activated silica, and 1.0 g Na2SO4 was done from the base. Five (5) mL of hexane was used to condition the packed column. The extract was poured into the column and eluted with 10 mL of n-hexane. Three hundred microliters of analytical grade nonane was employed to spike the eluate and was concentrated to about a 1–2 mL volume by passing through a mild stream of nitrogen (99.99%). The obtained samples were kept in colored vials and refrigerated prior to final GC analysis.

Quantification of PBDEs was carried out using a Trace 1300 Series GC fixed to a TSQ8000 Mass Spectrometer operated in selected reaction monitoring (SRM) mode with the electron impact (EI) ionization method. A ZB 274305 column (30 m length, 0.25 mm i.d., 0.25 mm film thickness) was used for separation and quantification of the analytes. The carrier gas employed was high purity helium gas with an optimum flow rate of 1.0 mL/min during regular flow mode; and nitrogen gas as a make-up gas at a flow rate of 20 mL/min. The temperature of the injector was set at 280 °C; and the detector set at 320 °C. The temperature of the oven was set at 150 °C and held for 2 min, ramped at 8 °C/min to 320 °C, and was held for 2 min. Using the splitless injection mode, 1 µL each of the mixed standard solutions and extracts were injected into the GC.

The quantification of all the targeted PBDE compounds was based on peak areas using external calibration curve techniques. Construction of the calibration plots was done by using six calibration points of the PBDE congeners. The retention times of each of the analytes were compared with that of the reference standards. The peak identification was based on the base ions and the isotope pattern of each PBDE compound in the MS/MS spectra. Base ions were selected as quantitative ions, while the other ions were used as confirmatory ions (Table 1).

Amber colored bottles were chosen for water sampling and storage with the purpose of preventing photo degradation. Regular injection of a solvent blank was observed for maximum performance of the GC, and the determination of procedural blanks was also done. Preparation and instrumental determination of the standards of the PBDE compounds were carried out to make sure that the detector responded to the PBDE compounds. Spiking of the matrix was also observed. Moreover, a calibration check of a 5 ng/mL standard was carried out after every five sample runs. This was to make certain that less than twenty percent disparity was found from the early calibration standards.

Recovery analysis was carried out in which a 1000 mL aliquot of acidified millipore distilled water (18.2 µm) was transferred to a 2000 mL separatory funnel and was then spiked with 10 mL of 1000 ppb standard mixture consisting of six different PBDEs congeners. The mixture was thoroughly shaken together to ensure an even distribution. After equilibration, the sample water was extracted with 10 mL of 1000 ppb mixture of the PBDEs, in spectroscopic grade hexane followed by clean-up. The extracted samples were placed in an oven dried amber bottle. The samples were then dried under nitrogen gas and re-dissolved in 1 mL n-hexane. The GC was injected with 1 µL of the mixture. The amount of the analytes recovered was determined and the percent recovery was estimated by comparison of the peak areas of the PBDEs following spiking with that of the evaporated standard residues. Both pentachloronitrobenzene (PCNB) and BDE 77 were employed as substitute standards to assess the recoveries of the six PBDEs compounds in the water sample. The recovery experiment carried out showed that the PBDEs compounds ranged between 105% (BDEs 100 and 153) and 110% (BDE 28) and the substitute standards ranged between 95% (BDE 77) and 103% (PCNB). The calibration plot for the individual PBDE congeners was linear with a correlation coefficient (r^2^) of 0.9914. Table 1 shows the retention times and (*m*/*z*) of the target analytes.

### 2.4. Statistical Analysis of the Data

Processing of the data was done with a 2007 Microsoft Excel file. Statistical Package for the Social Science (SPSS) version 15 for window evaluation was employed for the analysis of the data. Descriptive and inferential statistics such as one-way ANOVA and Duncan analysis were carried out to determine significant differences between mean values. In order to estimate the degree of association among the PBDE compounds, Pearson Correlation (two-tailed) was employed.

## 3. Results and Discussion

### 3.1. Concentrations of PBDEs in Stream Water

Table 2 presents the monthly variations of PBDEs in the water samples collected from six sampling sites. No data was obtained for December because all the locations investigated in this study were completely dried up during that month. The mean concentration of the Ʃ_6_PBDEs in the water ranged between 0.03 and 0.45 ng/mL. This result is lower when compared to the values recorded by [3] for Diep River, Cape Town, South Africa with ranges from 0.84 to 10,900, 4.99 to 12,000, and 2.89 to 569 ng/mL, while values for the upstream, point of discharge, and downstream locations in the Kuils River, South Africa ranged from 0.71 to 18,500, 22.5 to 176, and 23.6 to 403 ng/mL, respectively.

The highest mean Ʃ_6_PBDEs value (0.45 ng/mL) was obtained in July followed by 0.18 ng/mL in June, while the lowest mean value of 0.03 ng/mL was recorded in November. The higher levels of PBDEs recorded during the rainy season might be due to active mobilization of leached PBDE congeners by erosion into the water body, while the low mean value of 0.06 ng/mL recorded in August could be due to the August rainfall break.

The ANOVA analysis of these data indicated that all the PBDE compounds were significantly different (*p* < 0.05) from month to month throughout the sampling time. This is a clear indication of the non-uniformity in the discharge of the PBDE compounds into the water body. Correspondingly, observations revealed that there existed considerable differences (*p* < 0.05) in the concentrations of all of the congeners in the water body from one sampling period to another. This implies that variations in the climate condition at different seasons of the year could significantly affect the levels of these contaminants in a given environmental compartment.

The Duncan Multiple range test was also carried out on the data to ascertain likely differences in the PBDEs’ mean values in each of the sampling periods, and it was shown that nearly all of the congeners were not statistically different except for BDEs 28 and 153. This could be an indication of related types of waste dumped in this dumpsite over time. The PBDE congeners analyzed in this study indicated that all technical products used as standards were present in the water samples.

The concentrations of PBDE congeners at the six locations along the water course were evaluated to assess their variation patterns. The results obtained for the various locations are presented in Table 3. The findings confirmed that PBDEs were mobile and could be transported over a long distance over time. Moreover, all the PBDE congeners analyzed were present in all of the locations at varying concentrations. Spatially, the mean levels of the total PBDEs ranged between 0.03 and 0.31 ng/mL.

The mean concentrations of BDE 153 appeared to be the highest of all the congeners evaluated. Daso et al. (2013) [3] reported BDE 47 as the most dominant congener in water samples from the Diep River, Cape Town, South Africa. Moreover, Hallgreen et al. (2001) [32] reported that BDE 47 is generally reported as the most abundant congener in environmental water samples worldwide. The vegetation cover in the Asunle stream was about 90% and exposure of the stream to sunlight was very low, hence, debromination occured. This could be a possible reason for the dominance of this congener in the stream since sunlight causes debromination of lower PBDE congeners. The observed trend in PBDE levels was such that the mean concentration obtained at location 1 was significantly higher than all of the other locations. This was apparently due to the close proximity of this location to the dumpsite where the leaching of the contaminants into the receiving stream would be expected to be the most intense. The mean concentration (0.23 ng/mL) at the upstream point was very close to that of location 1 (0.31 ng/mL) although the upstream location was uphill, 0.25 km distance from the dumpsite. The source of these contaminants at location 0 could be attributed to atmospheric deposition resulting from frequent open incineration of solid wastes at the dumpsite. This confirmed that the deposition from the atmosphere is a vital medium of transport of particulate contaminants to the ecosystem [33,34].

Generally, from location 1, the mean concentration of these contaminants decreased downstream probably as a result of dilution effects as the stream volume increased downstream. However, the mean concentration of the contaminants at location 5 was slightly higher than that at location 4. This could be due to the fact that location 5 had a lower topography with greater surface area and greater capacity to retain particulates as water flowed out of the point more gently than at location 4. The concentrations of BDEs 28 and 47 were distinctly much lower when compared with other higher congeners, especially BDE 153. This could be because the lower molecular weight brominated congeners easily volatilize and are more readily soluble in water, in agreement with the study by Palm et al. [35].

**Figure 2:**
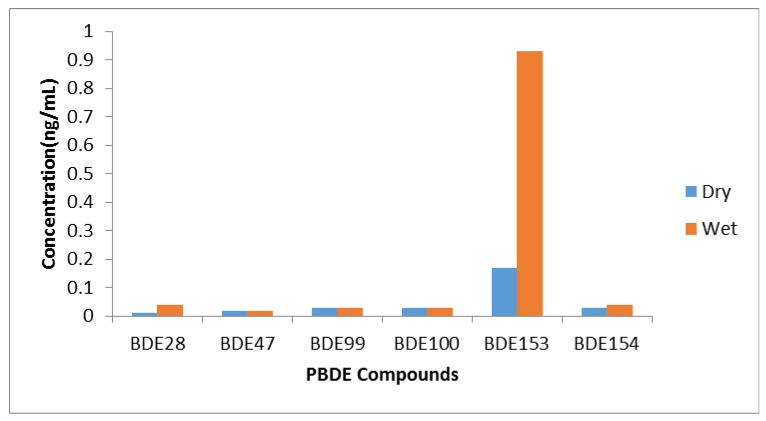
Seasonal Variation in the Levels of PBDE Compounds in the Asunle Stream Water Samples.

Figure 2 presents the seasonal variation in the levels of PBDE compounds in water samples from the Asunle stream. The concentrations (ng/L) of these congeners for the dry and wet seasons are as follows: BDE 28 (0.01, 0.04); BDE 47 (0.02, 0.02); BDEs 99 and 100 (0.03, 0.03); BDE 153 (0.17, 0.93); and BDE 154 (0.03, 0.04), respectively. The concentrations of the BDE 28 and BDE 153 congeners showed significant differences in the wet season in the stream water, but the value obtained for BDE 153 during the wet season was more pronounced. The predominance of BDE 153 during the wet season could be due to the possible high technical formulation of the BDE 153 congener present in the waste. Furthermore, the high vegetation cover (90%) of the stream could hinder the penetration of sunlight thereby preventing the debromination of the lower PBDE congeners thus resulting in the dominance of this congener. The possible influence of PBDE compounds leached from the dumpsite as a result of heavy precipitation and subsequent discharge of runoff into the stream might be a strong factor that led to the high levels of these contaminants being recorded during the wet season.

Table 4 presents the two-tailed correlation breakdown of PBDEs in the water samples. From Table 4, it could be seen that BDEs 47, 153, and 99 showed positive correlations with BDE 28 at *p* < 0.05 and *p* < 0.01 levels, correspondingly; BDEs 100 and 153 positively correlated with BDE 99 at *p* < 0.05; and *p* < 0.01 levels while BDE 154 was negatively correlated with BDEs 99, 100, and 153. These positive correlations could be suggestive of the same source which might be from end users of goods containing PBDEs dumped into the dumpsite from which the PBDEs seeped into the stream.

### 3.2. Comparison of PBDE Levels in Water Samples with Levels in Various Countries of the World

Comparing the results of this study to international concentrations of polybrominated diphenyl ethers in water with similar studies on the amount of PBDEs in the water bodies of many regions of the world is quite revealing. The levels of Σ_6_PBDEs in this research work ranged between 0.03 and 0.45 ng/L (Table 5), whereas the concentrations of water total PBDEs reported in different parts of the globe mostly ranged between <0.1 and 500 ng/mL apart from effluent samples from waste water treatment plants in California which had a sum level of PBDEs at 29,023 ng/mL [36].

Clearly, the PBDEs levels obtained from this research work were generally below the global values recorded elsewhere. The highest level of most of the PBDE compounds recorded at location 1 and at the downstream sampling points were below 5 ng/mL. Going by this trend, the overall contribution of these contaminants to the pollution status of the water body should not be left unchecked. This is because possible further and future accumulations of these contaminants in the water body could lead to serious health effects on the inhabitants of the communities that depend on this stream for their consumption and many other household uses.

The concentration of PBDEs recorded in the Asunle Stream could be due to contaminants transported into the stream through run-off, especially during periods of heavy rainfall. Eljarrat et al. (2008) [37] affirms that run-off immensely contributes to the re-distribution of PBDEs released from soil and land to which PBDEs had been previously applied. With the increasing usage of this water for drinking and processing of farm produce by the farmers and rural settlers along the stream, there is a need for conscious steps to be taken to assess the possible impacts of the concentrations of these contaminants in the water body on the public health. There is the possibility of bioaccumulation and biomagnification of the contaminants along the trophic levels of the aquatic organisms in the stream under investigation. Equally important is the fact that there could be plant uptake of these contaminants and subsequent accumulation in plant tissues [38,39]. Consequently, it is very necessary to put proper procedures in place to check the additional release of PBDE compounds into the water body in order to forestall the permanent damage that could result from human exposure to these harmful contaminants by the rural dwellers that produce the food items consumed in towns and cities.

## 4. Conclusions

Despite the extensive works that have been done globally on persistent organic pollutants, there is a dearth of information about BFRs in the Nigerian environment. This study addresses a knowledge gap by providing data on the contamination status of PBDEs in a water body, an important contribution regarding environmental matters on new emerging contaminants.

The detection of PBDEs at all locations of the Asunle stream that were investigated showed that this pollutant is ubiquitous and capable of long range transport. The PBDEs levels recorded in this study, though relatively low, are of urgent concern because of the potential health impacts of PBDEs on villagers who depend on this stream for water supply for drinking, cooking, and other purposes. Hence, there is a need to put in place effective control methods that could mitigate the leaching of PBDEs from the dumpsite into the environmental compartments nearby.

The contribution of rainfall on the quantity of leachate formed in the dumpsites that found its way into the stream was most pronounced during the June/July sampling regimes. During these periods, the ∑_6_BDEs of these contaminants were very high compared to the other months. The results recorded in this research work showed the dominance of the BDE 153 congeners in all of the locations, particularly during the wet season. This could probably be a sign of the dominance of its technical formulation in the e-waste dumped in this dumpsite. A more comprehensive understanding would be achieved by further studies on the dynamic pollution mechanism and aquatic biota in this region. Study of the aquatic biota would provide an indication of the bioavailability of these new emerging contaminants.

## Figures and Tables

**Table 1:** Target Ions of Investigated PBDE Congeners Monitored by GC-MS/MS.

Analytes	BDE28	BDE 47	BDE100	BDE99	BDE153	BDE154	BDE77	PCNB
Retention time (min)	14.72	17.37	19.29	19.90	21.38	22.18	18.74	7.13
Quantitative ions (*m*/*z*)	405	487	404	404	485	485	485	294
Qualifier ions (*m*/*z*)	405, 247, 246	487, 327, 325	405, 137, 137	404, 297, 295	485, 483, 295	485, 485, 376	405, 325, 246	264, 248, 236, 176
Collision energy	16, 14	18, 16	30, 30, 10	32, 28	32, 32, 10	32, 10	NA	NA

NA = Not available.

**Table 2:** Mean Concentration (ng/mL) of Selected PBDEs in Water Samples Collected from Asunle Stream.

Month	BDE28	BDE47	BDE99	BDE100	BDE153	BDE154	Σ_6_PBDE
Nov.	Nd	0.02 ± 0.01	0.02 ± 0.02	0.01 ± 0.02	0.05 ± 0.08	0.06 ± 0.03	0.03
Jan.	0.03 ± 0.04	0.01 ± 0.02	0.05 ± 0.04	0.04 ± 0.06	0.35 ± 0.45	0.02 ± 0.02	0.08
Feb.	0.01 ± 0.02	0.02 ± 0.03	0.01 ± 0.01	0.04 ± 0.04	0.12 ± 0.24	0.02 ± 0.01	0.04
May.	0.03 ± 0.04	0.02 ± 0.02	0.03 ± 0.04	0.06 ± 0.06	0.23 ± 0.34	0.02 ± 0.04	0.07
Jun.	0.03 ± 0.05	0.03 ± 0.02	0.02 ± 0.04	Nd	0.79 ± 1.79 *	0.03 ± 0.04	0.18
Jul.	0.06 ± 0.05	0.03 ± 0.02	0.04 ± 0.04	0.04 ± 0.06	2.49 ± 3.76 *	0.03 ± 0.03	0.45
Aug.	0.03 ± 0.05	0.02 ± 0.03	0.02 ± 0.04	0.01 ± 0.03	0.23 ± 0.34	0.06 ± 0.02	0.06

Mean values with asterisk (*) are at *p* < 0.05 significantly different. Nd = not detected.

**Table 3:** Mean Levels (ng/mL) of the Spatial Allocation of PBDEs in the Asunle Stream Water Samples.

Location	BDE28	BDE47	BDE99	BDE100	BDE153	BDE154	Σ_6_PBDEs
**0**	0.01 ± 0.03	0.02 ± 0.02	0.02 ± 0.04	0.01 ± 0.03	1.31 ± 3.05	0.03 ± 0.04	0.23
**1**	0.05 ± 0.04	0.02 ± 0.01	0.03 ± 0.04	0.04 ± 0.05	1.65 ± 2.64	0.04 ± 0.04	0.31
**2**	0.02 ± 0.03	0.03 ± 0.02	0.03 ± 0.02	0.05 ± 0.06	0.24 ± 0.21	0.03 ± 0.02	0.07
**3**	0.04 ± 0.05	0.02 ± 0.03	0.04 ± 0.03	0.04 ± 0.03	0.20 ± 0.41	0.05 ± 0.03	0.07
**4**	0.02 ± 0.04	0.02 ± 0.01	0.01 ± 0.01	0.01 ± 0.03	0.06 ± 0.07	0.05 ± 0.03	0.03
**5**	0.01 ± 0.03	0.02 ± 0.02	0.04 ± 0.03	0.03 ± 0.05	0.18 ± 0.31	0.02 ± 0.03	0.05

**Table 4:** Correlation coefficient of PBDEs in the Asunle Stream Water Samples.

	BDE28	BDE47	BDE99	BDE100	BDE153	BDE154
BDE28	1.00	0.351 *	0.406 **	0.179	0.350 *	−0.117
BDE47		1.00	0.119	0.104	0.015	0.084
BDE99			1.00	0.392 *	0.514 **	−0.417 **
BDE100				1.00	0.163	−0.451 **
BDE153					1.00	−0.319 *
BDE154						1.00

Single (*) and double asterisk (**) indicate significant correlation at *p* < 0.05 and 0.01 levels; *n* = 240.

**Table 5:** Comparison of PBDE Levels in Water Samples with other Studies around the World

Location	ΣPBDEs ( tri-hept)	BDE 47	BDE 99	Reference
Mendoza River, Argentine	Nd-1.9 ^b^	-	-	[26]
Scheldt Estuary and North Sea, Dutch Coast	<0.1–5.6 ^a^	1	0.5	[40]
Waste Water Treatment Plant, California	29,023 ^a^	10,467	11,200	[41]
Lake Ontario, North America	4–13 ^a^	0.11–3.83	-	[42]
San Francisco Estuary, California	3–513 ^a^	<16.1–179.5	<13.1–90.7	[35]
Pearl River, South China	0.344–0.68 ^b^	3–143	<1–200	[43]
Leachate, Pretoria, South Africa	9.79 ^b^	-	-	[44]
Diep/Kuils Rivers, South Africa	0.25–21,200 ^b^	-	-	[45]
Lake Shihwa, Korea	1.1–11(creek) ^b^ 0.25–2.1(in shore) ^b^ 0.16–0.37 (off shore) ^b^ 0.1	-	-	[46]
Asunle Stream, Ile-Ife	0.03–0.45 ^a^	-	-	This study

a = (ng/mL), b = ng/L.
